# Performance Analysis Based on Sustainability Exergy Indicators of High-Temperature Proton Exchange Membrane Fuel Cell

**DOI:** 10.3390/ijms231710111

**Published:** 2022-09-04

**Authors:** Xinjia Guo, Bing Xu, Zheshu Ma, Yanju Li, Dongxu Li

**Affiliations:** College of Automobile and Traffic Engineering, Nanjing Forestry University, Nanjing 210037, China

**Keywords:** high-temperature proton exchange membrane fuel cell, exergy analysis, exergy sustainability indicators, exergy balance

## Abstract

Based on finite-time thermodynamics, an irreversible high-temperature proton exchange membrane fuel cell (HT-PEMFC) model is developed, and the mathematical expressions of exergy efficiency, exergy destruction index (EDI), and exergy sustainability indicators (ESI) of HT-PEMFC are derived. According to HT-PEMFC model, the influences of thermodynamic irreversibility on exergy sustainability of HT-PEMFC are researched under different operating parameters that include operating temperatures, inlet pressure, and current density. The results show that the higher operating temperature and inlet pressure of HT-PEMFCs is beneficial to performance improvement. In addition, the single cell performance gradually decreases with increasing current density due to the presence of the irreversibility of HT-PEMFC.

## 1. Introduction

In recent years, proton exchange membrane fuel cells (PEMFCs) have been regarded as clean and promising energy conversion devices [1,2,3,4,5,6]. PEMFC has been widely applied in the vehicle and aerospace fields because of its low noise, zero-emission, fast start-up, and high reliability [7,8,9,10]. PEMFC can be divided into low-temperature PEMFC (LT-PEMFC, 60–80 °C) and HT-PEMFC (120–200 °C) according to the operating temperature. Compared with LT-PEMFC, HT-PEMFC has the advantage of accelerated reaction kinetic at the electrode and heat management system [11,12,13,14,15], higher CO tolerance [16,17,18], and higher quality waste heat [19,20].

The present research on HT-PEMFC mainly included material [21,22,23,24] improvement and methods of preparation [25,26,27,28,29]. Araya et al. [30] reviewed the current status of research on the operational aspects and performance of HT-PEMFCs. It focused on phosphoric acid-doped polybenzimidazole (PBI)-based HT-PEMFCs and reviewed their single-cell, stack, and system-level designs. Techniques for cell health status diagnosis, cell failure prevention, and cell life extension are also discussed. Gao et al. [31] proposed a numerical model of a HT-PEMFC stack waste heat recovery system and verified the model based on experiments to determine the main variables before optimizing the system configuration to recover heat from the exhaust gas. The results showed that model accuracy and system configuration optimization are crucial. Esfeh et al. [32] established the PEMFC model and conducted a parametric study. The results showed that the temperature increase reduced the concentration loss in all the temperature ranges, but reduced the activation overpotential below 80 °C. Miansari et al. [33] investigated the effects of parameters such as pressure, temperature, anode, and cathode channel depth on the performance of PEMFCs. The results showed that increasing the operating temperature and pressure can improve the cell performance, exergy efficiency and reduce the irreversibility of the cell. Li et al. [34] established a model of irreversible proton exchange membrane fuel cell including polarization loss and leakage current loss based on finite time thermodynamics. According to the model, the influence of the operating temperature, operating pressure, and water content of the proton exchange membrane on the optimal performance of the irreversible proton exchange membrane fuel cell is investigated. The results showed that when the operating temperature is increased, the optimized performance of the proton exchange membrane fuel cell in terms of output power, output efficiency, ecological objective function, and ecological coefficient of performance will be improved.

Guo et al. [35] researched the power, exergy, and ecological analysis of HT-PEMFC based on a phosphoric acid-doped polybenzimidazole membrane, and the experimental results showed that the operating temperature and doping level have a greater impact on improving the performance of HT-PEMFC than the operating pressure and relative humidity. Khan et al. [36] proposed a semi-empirical model of HT-PEMFC, taking into account hydrogen pressure, ambient temperature, pressure, and load resistance, and researched the influence of these parameters on cell performance. The results showed that when the ambient temperature increases and the pressure decreases, the output voltage of HT-PEMFC decreases. Nalbant et al. [37] researched the exergy performance evaluation of an integrated CHP system based on HT-PEMFC. The results showed that when the operating temperature of HT-PEMFC increases, the power and CHP efficiency of the system improved, and the stoichiometric ratio of the anode is the most influential parameter on system performance.

Toghyanni et al. [38] conducted an economic analysis on the energy efficiency of HT-PEMFC by using a 3D non-isothermal model and researched the influences of operating temperature, cathode pressure, the thickness of gas diffusion layer, and membrane on the exergy efficiency and exergy cost. The research results showed that when the operating temperature and thickness of the membrane increased from 363 K to 393 K and 50 μm to 183 μm, respectively, the exergy cost of hydrogen decreased exergy efficiency is slightly improved and the higher operating pressure will reduce the exergy cost of hydrogen. Ye et al. [39] researched exergy analysis of the HT-PEMFC system. The research results showed that the increasing the inlet relative humidity and pressure effects on the improvement of the two kinds of system performance were indistinctive, further enhancing the efficiency of the energy exchange of the fuel cell and reducing the irreversibility of fuel cells is the key point of further improve the system efficiency.

In recent years, PEMFC had made some progress in exergy-related research. Ay et al. [40] researched the changes of exergy efficiency under different working conditions. The research results showed that the exergy efficiency of PEMFC decreased with the increase of thickness of the membrane and current density. In the case of the same thickness of the membrane, the exergy efficiency increased with the increase of cell operating pressure and the decrease of current density. Therefore, in order to improve the performance of PEMFC, the lower thickness of the membrane, lower current density, and higher cell operating pressure should be selected under the condition of constant cell temperature. Li et al. [41] researched exergy performance analysis and optimization of HT-PEMFC. The research results showed that increasing the inlet pressure and doping level can improve energy efficiency. Midilli et al. [42] proposed some new exergy-based indicators for PEMFC research through research, which are expected to quantify the sustainable concept of PEMFC.

In this paper, firstly, the irreversibility of the HT-PEMFC is analyzed according to finite time thermodynamics. A mathematical model that considers the irreversible polarization loss and leakage current loss is established. Secondly, exergy analysis is carried out on the HT-PEMFC operation process to research the exergy sustainability index of HT-PEMFC under different operating parameters. At the same time, the influences of different operating temperatures, the thickness of the membrane, and the current density on exergy sustainability index and cell operation process are researched. The results of the parametric study can provide directions for future improvements in the operating conditions and design of HT-PEMFC.

## 2. Results and Analysis

The relevant parameters in the HT-PEMFC model are shown in Table 1. The thermodynamic irreversibility and exergy sustainability of HT-PEMFC with different operating parameters are studied according to the input parameters.

**Figure 1:**
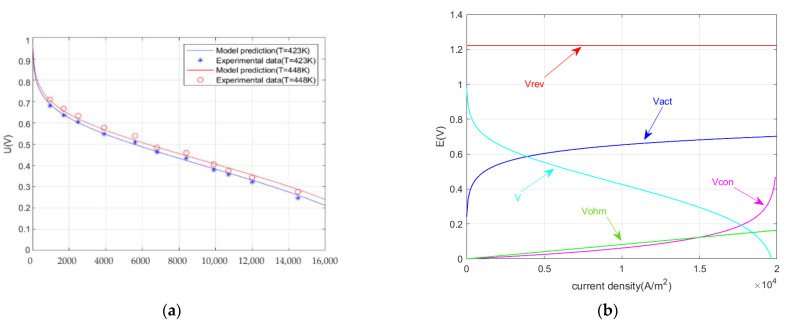
(**a**) Comparisons of the predicted model potential and the experimental data; (**b**) Curves of the potential and output voltage versus the current density.

Figure 1a compared the predicted model potential and experimental data [43] of HT-PEMFC at 423 K and 448 K (P = 1 atm; RH= 0.38%; DL = 5.6). As can be seen from Figure 1a, the error between the predicted and experimental data is about 6%. This shows that the model has a relatively good accuracy. Figure 1b shows the reversible potential, the polarization loss potential, and the output voltage versus the current density. It can be seen that the reversible potential is a constant that is unrelated to the current density. All three kinds of overpotentials increase with the increase of current density, where the concentration overpotential increases exponentially, the activation overpotential increases logarithmically, and the ohmic overpotential increases less. In the low current density section, the rapid growth of the activation overpotential leads to a decrease in the output voltage. In the higher current density section, the output voltage decreases mainly due to the rapid increase in the concentration overpotential.

**Figure 2:**
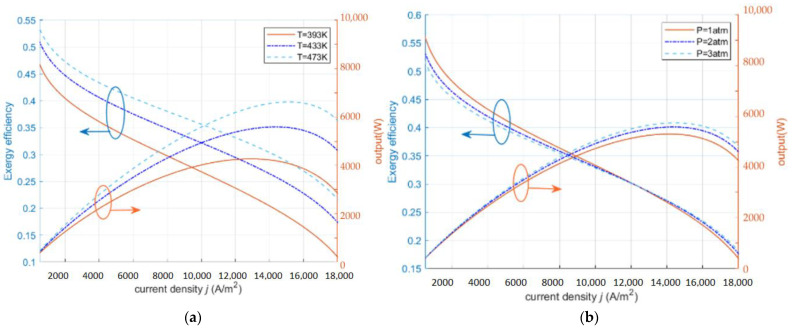
The effect of operating parameters on exergy efficiency and power density (RH=0.038%, $t_{mem}=0.002\;\mathrm{cm},DL=10$). (**a**) different T; (**b**) different P.

As shown in Figure 2a, exergy efficiency and power density increase slightly with the increase of operating temperature. From the perspective of electrode reaction kinetics, the increase of temperature is beneficial to increasing the proton conductivity and largely reduces the irreversible loss due to the polarization phenomenon. From the molecular viewpoint, the increase of temperature is beneficial to speed up the rate of proton motion and reaction, shorten the proton transport time, and thus improve the performance of HT-PEMFC. In the low current density section, the exergy efficiency stays at a high level and decreases when the current density increases to the higher section. In addition, the corresponding efficiency and power density do not reach the maximum value at the same moment when the power density or the exergy efficiency obtain the maximum value, respectively. In Figure 2b, both the power density and exergy efficiency increase significantly with the increase of the inlet pressure. When the inlet pressure increases, the gas concentration also increases, which improves the gas transport inside the fuel cell and makes the kinetic performance of the fuel cell improved. In the low current density interval, increasing the pressure is not obvious to improve the output performance of the fuel cell, but will cause an increase in the parasitic power due to the increase in the power of the air compressor, thus causing a decrease in system efficiency; in the medium current density interval, the effect of increasing the pressure on the output performance is gradually improved, gradually offsetting, or even higher than the parasitic power consumption that is brought by the air compressor; in the high current density interval, the higher the pressure, the higher the obstruction of protons through the membrane. The higher the pressure, the smaller the obstruction of protons through the membrane, and the mass transfer conditions are improved, leading to the reduction of ohmic loss, and the better the output performance of the fuel cell system.

### 2.1. Exergy Destruction Index (EDI)

**Figure 3:**
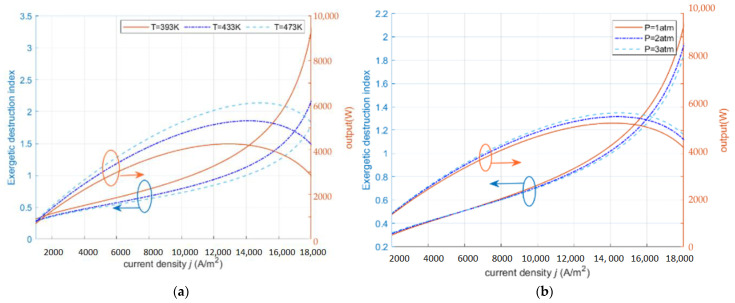
The effect of operating parameters on exergy destruction index and power density (RH=0.038%, $t_{mem}=0.002\;\mathrm{cm},DL=10$). (**a**) different T; (**b**) different P.

Figure 3a,b show the effects of operating temperature and inlet pressure on EDI. In Figure 3a, the EDI  decreases with the increase of the operating temperature when the operating temperature increases. From the energy balance point of view, the increase in operating temperature improves the operating environment of the fuel cell and reduces the irreversible exergy loss due to polarization losses, which makes the HT-PEMFC have less impact on the environment and therefore the EDI  is reduced. In Figure 3b, the increase in the inlet pressure leads to an increase in the EDI. The increase in the inlet pressure leads to a subsequent increase in the gas concentration, which improves the gas transport conditions inside the fuel cell and reduces the hindrance of the fuel cell in the electrochemical reaction. In addition, the increase of EDI with the temperature increase is relatively large. In the low current density section, the enhancement of EDI with temperature and pressure is relatively small, while in the high current density section, EDI is more affected by temperature. According to Equations (26), (28), (30), and (31), the HT-PEMFC at high current density is in the operation state of high output power and low assembly efficiency, which is a waste of energy and not environmentally friendly.

### 2.2. Exergy Sustainability Index (ESI)

**Figure 4:**
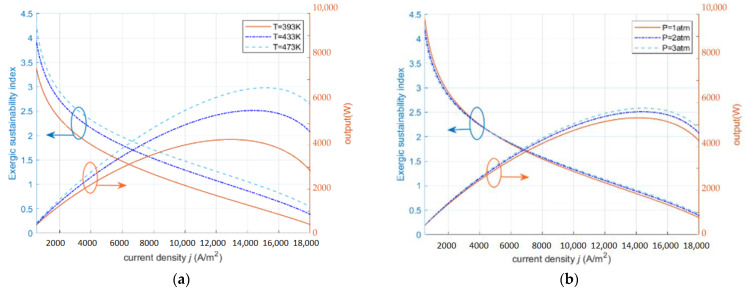
The effect of operating parameters on the exergy sustainability index and power density (RH=0.038, $t_{mem}=0.002\;\mathrm{cm},DL=10$). (**a**) different *T*; (**b**) different P.

In Figure 4a, ESI decreases with increasing current density. When the temperature is low, the activation loss of the HT-PEMFC is higher, and the resulting exergy loss and exergy dissipation are higher. When the temperature increases, the catalyst activity in the HT-PEMFC increases, the activation loss decreases, the output voltage increases, and the output performance of the fuel cell is improved. In Figure 4b, as the inlet pressure increases, increasing the pressure causes a slight decrease in the ESI at low current density, because the exergy loss and exergy dissipation due to parasitic power consumption from the air compressor are higher than the output performance improvement at this point. In the medium current density section and in the high current density section, increasing the inlet pressure and increasing the gas concentration, the output performance of the HT-PEMFC is improved at this time, and the ESI increases with the increase in the inlet pressure.

### 2.3. Exergy Sustainability Indicators

**Figure 5:**
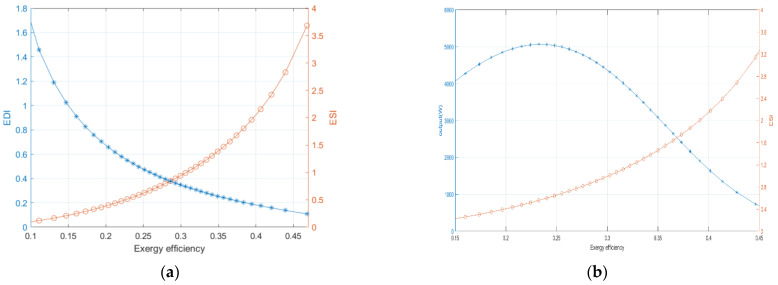
(**a**) Relationship between ESI and EDI at different exergy efficiency; (**b**) Relationship between ESI and P at different exergy efficiency.

Figure 5a,b show the curves of ESI,EDI, $\eta_{ex}^{fc}$ and net output power at the thickness of membrane of 0.002 cm, operating pressure of 3.039 × 10^5^ Pa, the operating temperature of 413 K and current density of 0–2 × 10^4^ A/m^2^. As the exergy efficiency increases, the EDI gradually decreases and the ESI increases. This indicates that the damage to the environment of the cell is gradually reduced when the HT-PEMFC continued to operate and the operating temperature is increasing until it is stable and also verifies the sustainability of the HT-PEMFC. As can be seen in Figure 5b, when the exergy efficiency and ESI increase, the output power density does not keep increasing continuously, which indicates that the best performance of HT-PEMFC requires us to further balance the indicators and perform multi-objective optimization of each indicator to obtain the optimal output power.

## 3. Thermodynamic Model

### 3.1. Working Principle of HT-PEMFC

The reaction principle of PEMFC is as follows: the hydrogen tank supplies hydrogen to the anode, and the air compressor supplies oxygen to the cathode. Then, the hydrogen is converted to hydrogen ions and electrons in the anode, the hydrogen ions pass through the proton exchange membrane, and the electrons flow through the external load to the cathode, where the hydrogen ions and oxygen atoms and electrons combine to form H_2_0 [44]. The electrochemical reaction formula of PEMFC is as follows:

(1) $$\mathrm{anode}\;\mathrm{reaction}:{\;H}_{2}\to 2H^{+}+2e^{-}$$

(2) $$\mathrm{Cathodic}\;\mathrm{reaction}:\;2H^{+}+\frac{1}{2}O_{2}+2e^{-}\to H_{2}O+\mathrm{heat}$$

(3) $$\mathrm{Total}\;\mathrm{reaction}:{\;H}_{2}+\frac{1}{2}O_{2}\to H_{2}O+\mathrm{heat}+\mathrm{electricity}$$

In HT-PEMFC, phosphoric acid is usually used instead of water for humidification. The principle of mass transfer in the anode, cathode, and membrane can be expressed as:

(4) $$\mathrm{anode}\;\mathrm{reaction}:{\;H}_{2}{\mathrm{PO}}_{4}^{-}+H^{+}=H_{3}{\mathrm{PO}}_{4}$$

(5) $$\mathrm{membrane}:{\;H}_{3}{\mathrm{PO}}_{4}+\mathrm{PBI}=H_{2}{\mathrm{PO}}_{4}^{-}+\mathrm{PBI}\cdot H^{+}$$

(6) $$\mathrm{Cathodic}\;\mathrm{reaction}:\;\mathrm{PBI}\cdot H^{+}=\mathrm{PBI}+H^{+}$$

### 3.2. Reversible Potential of HT-PEMFC

For exergy analysis and research, the changes of exergy sustainability indexes under different working conditions, the following assumptions should be considered for HT-PEMFC:

1. The fuel cell operates in a steady state.
2. The reactants are ideally compressible gases and there are no reactants left after the full reaction.
3. The operating pressure and relative humidity are constant during the reaction.
4. The effect of the generated water on the relative humidity is not considered.
5. Only physical and chemical exergy is considered, no potential exergy and kinetic exergy are considered.
6. Leakage current loss and polarization loss are considered.

In a HT-PEMFC thermodynamic system, the potential of an electrochemical reaction depends on the Gibbs free energy of fuel [44]. The operating temperature, pressure, and concentration of the reaction gas affect the Gibbs free energy. For HT-PEMFC, the reversible output voltage is as follows:

(7) $$V_{rev}=E_{r}^{0}+\frac{-18.449-0.01283T}{F}\left(T-T_{0}\right)+\frac{RT}{nF}\ln(\frac{p_{H_{2}}p_{O_{2}}^{0.5}}{p_{w}})$$

where $E_{r}^{0}=1.185$ is the reversible potential, T is the operating temperature of HT-PEMFC, $T_{0}$ is the ambient temperature, R is the gas constant, $p_{H_{2}}$ is the inlet pressure of hydrogen, $p_{O_{2}}$ is the inlet pressure of oxygen, and $p_{w}$ is the pressure of water vapor discharge.

### 3.3. Overpotential of HT-PEMFC

The HT-PEMFC shows polarization phenomena due to its electrochemical reactions and internal resistance. The polarization phenomenon means that during the electrochemical process of HT-PEMFC, energy must be consumed to overcome the resistance, such as the diffusion of anode and cathode gases and the dissolution of active gases, such as proton adsorption reactions. This energy consumption will reduce the actual output voltage and the electrode potential will be lower than the ideal reversible potential. Polarization phenomena cause three types of polarization overpotentials: activation overpotential, ohmic overpotential and concentration overpotential.

The electrochemical reaction rate of HT-PEMFC affects the activation over potential. The slower the reaction rate, the more severe the polarization loss and the greater the activation overpotential. In addition, the catalysts used at the cathode and anode poles are also related to the activated overpotential. The better the catalyst activity, the lower the activation overpotential. The activation overpotential $V_{act}$ can be expressed as followed [45]:

(8) $$V_{act}=\frac{RT}{2\alpha F}\ln(\frac{I+I_{leak}}{I_{0}})$$

where $\;I_{leak}$ is the leakage current density, $I_{0}$ is the exchange current density, and α is the transfer coefficient. The expression of $I_{0}$ is as follows:

(9) $$\ln(I_{0})=2.2266\times \frac{1000}{T}-0.4959$$

For HT-PEMFC, the loss due to resistance between cell components is called ohmic overpotential. Ohmic resistance consists of two main parts: resistance that is caused by the ion flow through the proton exchange membrane in the electrolyte and resistance that is caused by the flow of electrons through the electrode ends.

The ohmic potential $V_{ohm}$ can be expressed as follows [46]:

(10) $$V_{ohm}=I\left(\frac{l_{m}}{K_{m}}+\frac{2l_{d}}{\sigma_{d}}\right)$$

where $l_{m}$ is the thickness of the membrane, $K_{m}$ is proton conductivity, $l_{d}$ is the thickness of the diffusion layer, and $\sigma_{d}$ is the electron conductivity.

Proton conductivity $K_{m}$ needs to consider the influence of cell operating temperature, phosphoric acid doping level of the membrane, and the relative humidity, and its expression is as follows [47]:

(11) $$K_{m}=\frac{a\times b}{T}\times exp^{\frac{-c}{RT}}$$

(12) $$a=68DL^{3}-6324DL^{2}+65750\mathrm{DL}+8460$$

(13) $$b=\{\begin{array}{c} 1+\left(0.01704T-4.767\right)RH,\;373.15K\leq T\leq 413.15K\\ 1+\left(0.1432T-56.89\right)RH,\;413.15K<T\leq 453.15K\\ 1+\left(0.7T-309.2\right)RH,\;453.15K<T\leq 473.15K \end{array}$$

(14) c=−619.6DL+21750

where DL is the doping level of phosphoric acid in the proton exchange membrane and RH is the relative humidity of the reaction gas [48].

During the operation of HT-PEMFC, when the reaction gas is not provided in time, the electrode reaction surface cannot maintain the concentration of the reaction gas and concentration polarization will occur.

The expression of concentration overpotential $V_{conc}$ is as follows [49]:

(15) $$V_{conc}=\left(1+\frac{1}{\alpha }\right)\frac{RT}{nF}\ln\frac{I_{L}}{I_{L}-I}$$

where $I_{L}$ is the limiting current density [46].

The irreversible output voltage V of HT-PEMFC can be expressed as [50]:

(16) $$V=V_{rev}-V_{act}-V_{ohm}-V_{conc}\;=1.185-\left(1.91\times 10^{-4}+1.33\times 10^{-7}T\right)\left(T-298.15\right)+4.13\;\times 10^{-5}T\ln\frac{P_{H_{2}}{P_{O_{2}}}^{0.5}}{0.0243}-1.72\times 10^{-5}\times \;T\ln\frac{I+88458.17\cdot exp^{\frac{-2342.9}{T}}}{3.95\times 10^{-6}T^{3}-0.00424T^{2}+1.523T-183}\;-I\left(\frac{l_{m}T}{a\cdot b\cdot exp^{\frac{74.5\mathrm{DL}-2616}{T}}}+9.8\times 10^{-6}\right)$$

### 3.4. Exergy Balance Model

**Figure 6:**
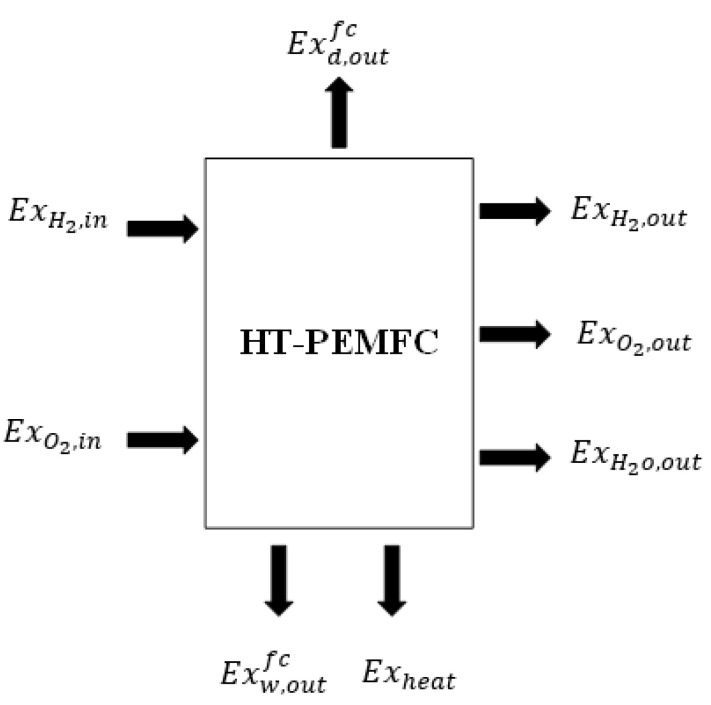
Exergy balance of proton exchange membrane fuel cell.

Considering the above assumptions, the exergy equilibrium model of HT-PEMFC is shown in Figure 6. The exergy balance expression of HT-PEMFC can be obtained by [51,52,53]:

(17) $$Ex_{in}^{fc}=Ex_{d,out}^{fc}+Ex_{w,out}^{fc}+Ex_{d}^{fc}$$

In the proton exchange membrane fuel cell system, the total exergy input is:

(18) $$Ex_{in}^{fc}=Ex_{H_{2},in}+Ex_{O_{2},in}$$

In this paper, exergy waste that is generated by hydrogen, oxygen, water, and heat that is discharged from the system can be divided into two categories: recoverable exergy waste and unrecoverable exergy waste [54].

(19) $$Ex_{w,out}^{fc}=Ex_{rw}^{fc}+Ex_{uw}^{fc}$$

The recoverable exergy waste is:

(20) $$Ex_{rw}^{fc}=n_{O_{2},out}\times {\left(ex\right)}_{O_{2}}^{ch}+n_{O_{2},out}\times {\left(ex\right)}_{O_{2}}^{ch}$$

The unrecoverable exergy waste is:

(21) $$Ex_{uw}^{fc}=\left(n_{H_{2},out}\times {\left(ex\right)}_{H_{2}}^{ph}+n_{O_{2},out}\times {\left(ex\right)}_{O_{2}}^{ph}+n_{H_{2}O,out}\times ex_{H_{2}O}^{ph}\right)\;+Q_{w,out}^{fc}\times \left(1-\frac{T_{0}}{T}\right)\times r_{hl}$$

Physical exergy and chemical exergy are expressed as followed [55]:

(22) $${\left(ex\right)}^{ph}=C_{p}T_{0}[\frac{T}{T_{0}}-1-\ln\left(\frac{T}{T_{0}}\right)+\ln\left(\frac{p}{p_{0}}{)}^{\frac{k-1}{k}}\right]$$

(23) $${\left(ex\right)}^{ch}=\sum x_{n}\cdot e_{n}^{ch}+RT_{0}\sum x_{n}\cdot \ln x_{n}$$

where $C_{p}$ is the constant specific heat of the gas, $T_{0}$ is the ambient temperature, $p_{0}$ is the pressure, K is the specific heat rate, $x_{n}$ is the mole fraction of the component, and $e_{n}^{ch}$ is the chemical exergy of each composition.

The total exergy of the desired output is:

(24) $$Ex_{d,out}^{fc}=W_{fc}$$

(25) $$W_{fc}=Vi=(V_{rev}-V_{act}-V_{ohm}-V_{conc})i$$

Exergy dissipation can be obtained from the equilibrium equation of PEMFC exergy

(26) $$Ex_{d}^{fc}=Ex_{in}^{fc}-Ex_{d,out}^{fc}-Ex_{w,out}^{fc}$$

### 3.5. HT-PEMFC Exergy Sustainability Index Derivation Process

The exergy efficiency of HT-PEMFC is defined as the ratio of effective exergy output to total exergy output, as follows [56]:

(27) $$\eta_{ex}^{fc}=\frac{Ex_{d,out}^{fc}}{Ex_{in}^{fc}}$$

Exergy dissipation will occur during HT-PEMFC operation due to heat transfer and polarization. The waste exergy ratio expression is as follows:

(28) $$r_{we}^{fc}=r_{rw}^{fc}+r_{uw}^{fc}\;r_{rw}^{fc}=\frac{Ex_{rw}^{fc}}{Ex_{in}^{fc}}$$

(29) $$\;r_{uw}^{fc}=\frac{Ex_{uw}^{fc}}{Ex_{in}^{fc}}$$

The irreversibility of HT-PEMFC during operation will lead to the increase of exergy dissipation. Exergy dissipation is used to describe the irreversibility of the operation process and the exergy dissipation rate is defined as:

(30) $$f_{exd}^{fc}=\frac{Ex_{d}^{fc}}{Ex_{in}^{fc}}$$

The exergy destruction index (EDI) can reflect the impact of unrecoverable exergy loss and exergy dissipation on the environment. Its expression is as follows:

(31) $$EDI=(r_{uw}^{fc}+f_{exd}^{fc})\;\frac{1}{\eta_{ex}^{fc}}$$

According to the reaction equation of PEMFC, the reaction products are only water, heat, and electric energy. In practical application, due to the irreversibility of cells, some $H_{2}$ and $O_{2}$ will not be used, which reduces the exergy stability of PEMFC, which is the main reason for exergy dissipation. In addition, power output also affects exergy stability. Therefore, the exergy stability coefficient of PEMFC is defined as:

(32) $$f_{est}^{fc}=\frac{Ex_{d,out}^{fc}}{\left(Ex_{d,out}^{fc}+Ex_{w,out}^{H_{2}}+Ex_{w,out}^{O_{2}}+Ex_{d}^{fc}\right)}$$

The Environmental Benign Index (EBI) indicates the environmental suitability of HT-PEMFC. The higher the *EBI* of HT-PEMFC, the better it is for the environment. The environmental adaptability of HT-PEMFC can generally be improved by reducing EDI. Its expression is as follows:

(33) $$EBI=\frac{\eta_{ex}^{fc}}{(r_{uw}^{fc}+f_{exd}^{fc})\;}$$

The exergy sustainability index (ESI) is as follows:

(34) $$ESI=\frac{\eta_{ex}^{fc}}{(r_{uw}^{fc}+f_{exd}^{fc})\;}\times \frac{Ex_{d,out}^{fc}}{\left(Ex_{d,out}^{fc}+Ex_{w,out}^{H_{2}}+Ex_{w,out}^{O_{2}}+Ex_{d}^{fc}\right)}$$

## 4. Conclusions

In this paper, a HT-PEMFC model is established based on finite time thermodynamics, which takes into account polarization losses and leakage current loss. In order to improve the sustainability of HT-PEMFC and reduce the negative impact on the environment, the exergy sustainability index of HT-PEMFC is studied to provide improvement directions for PEMFC in engineering applications. Some main conclusions are drawn through graphical and theoretical analysis as follows:

1. The reliability of the HT-PEMFC model is proved by comparing the model with the experimental data. Through the parameterization studies, the suitable increase of temperature is beneficial to the improvement of HT-PEMFC output performance. With the decrease of the plasmonic membrane thickness, the output performance is improved.
2. At low exergy efficiency, the output power of HT-PEMFC takes the maximum value and starts to decrease when the exergy efficiency exceeds 0.27.
3. The operating conditions of HT-PEMFC can be improved by increasing the inlet pressure, changing the diffusion rate of the gas, appropriately increasing the operating temperature, and using thin proton exchange membranes.

The obtainable conclusion may provide some directions and references for future research that is related to the influence of parameters on HT-PEMFC performance. In the future, the optimization method for output performance of HT-PEMFC can be further researched.

## Figures and Tables

**Table 1:** Relevant data of HT-PEMFC.

Parameter	Value
$\mathrm{Current}\;\mathrm{Density},\;I\;({\mathrm{Am}}^{-2}$)	0–20,000 [35]
Operating Temperature, T (K)	393–473 [35]
$\mathrm{Intake}\;\mathrm{Pressure},\;P_{H_{2}},\;P_{O_{2}}\left(\mathrm{atm}\right)$	1–3 [35]
Electronic Number, n	2
$\mathrm{Faraday}\;\mathrm{Constant},\;F\;\left({\mathrm{Cmol}}^{-1}\right)$	96,485
$\mathrm{Ambient}\;\mathrm{Temperature},\;T_{0}\;$(K)	298.15
Transfer Coefficient,α	0.25 [35]
Gas Constant, R	8.3143

## Data Availability

Not applicable.

## References

[1] Zhang Z.G. (2020). Researches on Green Features and Category Architecture of Green Strategies of Renewable-Resource-Based Enterprises: A Case Study of Forestry Enterprise. J. Nanjing For. Univ. Nat. Sci. Ed..

[2] Liu S.Q., Jia L.M. (2020). Review on Sustainable Development of Forest-based Biodiesel. J. Nanjing For. Univ. Nat. Sci. Ed..

[3] Yan Z.X., Yang H.Y., Fan S.F., Wu W.L., Lyu L.F., Li W.L. (2022). Analysis of the Expression of Sucrose Phosphate Synthase Genes Duringthe Development of Blackberry Fruit. J. Nanjing For. Univ. Nat. Sci. Ed..

[4] Zhou W., Zheng Y., Pan Z., Lu Q. (2021). Review on the Battery Model and SOC Estimation Method. Processes.

[5] Zhou W., Lu Q., Zheng Y. (2022). Review on the Selection of Health Indicator for Lithium Ion Batteries. Machines.

[6] Cheng Z., Zhou H., Lu Z. (2022). A Novel 10-Parameter Motor Efficiency Model Based on I-SA and Its Comparative Application of Energy Utilization Efficiency in Different Driving Modes for Electric Tractor. Agriculture.

[7] Haider R., Wen Y., Ma Z.F., Wilkinson D.P., Zhang L., Yuan X., Song S., Zhang J. (2021). High Temperature Proton Exchange Membrane Fuel Cells: Progress in Advanced Materials and Key Technologies. Chem. Soc. Rev..

[8] Xie J.Y., Xu X., Cai B., Zhang H.G. (2022). Responses of Forest Soil Labile Nitrogen Pool and Nitrogen Cycle to the Changes of Carbon Input under Carbon Neutrality. J. Nanjing For. Univ. Nat. Sci. Ed..

[9] Sonne C., Xia C., Lam S.S. (2022). Is Engineered Wood China’s Way to Carbon Neutrality?. J. Bioresour. Bioprod..

[10] Zheng C., Xu D.C., Cao J., Li L., Wen B. (2021). Design of Lightweight Electric Forestry Monorail Vehicle. J. For. Eng..

[11] Alpaydin G.U., Devrim Y., Colpan C.O. (2019). Performance of an HT-PEMFC Having a Catalyst with Graphene and Multiwalled Carbon Nanotube Support. Int. J. Energy Res..

[12] Jörissen L., Garche J. (2015). Polymer Electrolyte Membrane Fuel Cells. Hydrog. Fuel Cell Technol. Mark. Perspect..

[13] Harikishan Reddy E., Jayanti S. (2012). Thermal Management Strategies for a 1 KWe Stack of a High Temperature Proton Exchange Membrane Fuel Cell. Appl. Therm. Eng..

[14] Wei L., Deng W., Li S., Wu Z., Cai J., Luo J. (2022). Sandwich-like Chitosan Porous Carbon Spheres/MXene Composite with High Specific Capacitance and Rate Performance for Supercapacitors. J. Bioresour. Bioprod..

[15] Lu Q., Zhou W., Zheng Y. (2022). Regenerative Braking Control Strategy with Real-Time Wavelet Transform for Composite Energy Buses. Machines.

[16] Reddy E.H., Monder D.S., Jayanti S. (2013). Parametric Study of an External Coolant System for a High Temperature Polymer Electrolyte Membrane Fuel Cell. Appl. Therm. Eng..

[17] Ewulonu C.M., Liu X., Wu M., Yong H. (2019). Lignin-Containing Cellulose Nanomaterials: A Promising New Nanomaterial for Numerous Applications. J. Bioresour. Bioprod..

[18] Yang H.Q., Yu Z.H. (2021). Research Trends and Future Key Issues of Global Harvested Wood Products Carbon Science. J. Nanjing For. Univ. Nat. Sci. Ed..

[19] Lee D., Lim J.W., Lee D.G. (2017). Cathode/Anode Integrated Composite Bipolar Plate for High-Temperature PEMFC. Compos. Struct..

[20] Ding L., Han X., Cao L., Chen Y., Ling Z., Han J., He S., Jiang S. (2021). Characterization of Natural Fiber from Manau Rattan (Calamus Manan) as a Potential Reinforcement for Polymer-Based Composites. J. Bioresour. Bioprod..

[21] Li Q.F., Rudbeck H.C., Chromik A., Jensen J.O., Pan C., Steenberg T., Calverley M., Bjerrum N.J., Kerres J. (2010). Properties, Degradation and High Temperature Fuel Cell Test of Different Types of PBI and PBI Blend Membranes. J. Memb. Sci..

[22] Oono Y., Sounai A., Hori M. (2009). Influence of the Phosphoric Acid-Doping Level in a Polybenzimidazole Membrane on the Cell Performance of High-Temperature Proton Exchange Membrane Fuel Cells. J. Power Sources.

[23] Pinar F.J., Cañizares P., Rodrigo M.A., Úbeda D., Lobato J. (2015). Long-Term Testing of a High-Temperature Proton Exchange Membrane Fuel Cell Short Stack Operated with Improved Polybenzimidazole-Based Composite Membranes. J. Power Sources.

[24] Ebrahimi M., Kujawski W., Fatyeyeva K., Kujawa J. (2021). A Review on Ionic Liquids-Based Membranes for Middle and High Temperature Polymer Electrolyte Membrane Fuel Cells (PEM FCs). Int. J. Mol. Sci..

[25] Jiao K., Xuan J., Du Q., Bao Z., Xie B., Wang B., Zhao Y., Fan L., Wang H., Hou Z. (2021). Designing the next Generation of Proton-Exchange Membrane Fuel Cells. Nature.

[26] Muthuraja P., Prakash S., Shanmugam V.M., Radhakrsihnan S., Manisankar P. (2018). Novel Perovskite Structured Calcium Titanate-PBI Composite Membranes for High-Temperature PEM Fuel Cells: Synthesis and Characterizations. Int. J. Hydrogen Energy.

[27] Ma C.H., Luo Y.H., Li J.H., Huang Y.X., Jiang N., Guo W.Q. (2021). Study on the Enrichment of Isoxaziridin from Acanthopanax Senticosus by Macroporous Resin Immobilized with Ionic Liquid. J. For. Eng..

[28] Ma C.H., Sun J.D., Li W., Luo S., Liu S.X. (2021). Application Progress of Ionic Liquids in the Field of Lignin Depolymerization. J. For. Eng..

[29] Chen M., Gao X. (2014). Theoretical, Experimental and Numerical Diagnose of Critical Power Point of Thermoelectric Generators. Energy.

[30] Araya S.S., Zhou F., Liso V., Sahlin S.L., Vang J.R., Thomas S., Gao X., Jeppesen C., Kær S.K. (2016). A Comprehensive Review of PBI-Based High Temperature PEM Fuel Cells. Int. J. Hydrogen Energy.

[31] Gao X., Andreasen S.J., Chen M., Kær S.K. (2012). Numerical Model of a Thermoelectric Generator with Compact Plate-Fin Heat Exchanger for High Temperature PEM Fuel Cell Exhaust Heat Recovery. Int. J. Hydrogen Energy.

[32] Esfeh H.K., Hamid M.K.A. (2014). Temperature Effect on Proton Exchange Membrane Fuel Cell Performance Part II: Parametric Study. Energy Procedia.

[33] Miansari M., Sedighi K., Amidpour M., Alizadeh E., Miansari M. (2009). Experimental and Thermodynamic Approach on Proton Exchange Membrane Fuel Cell Performance. J. Power Sources.

[34] Li C., Liu Y., Xu B., Ma Z. (2019). Finite Time Thermodynamic Optimization of an Irreversible Proton Exchange Membrane Fuel Cell for Vehicle Use. Processes.

[35] Guo Y., Guo X., Zhang H., Hou S. (2020). Energetic, Exergetic and Ecological Analyses of a High-Temperature Proton Exchange Membrane Fuel Cell Based on a Phosphoric-Acid-Doped Polybenzimidazole Membrane. Sustain. Energy Technol. Assess..

[36] Khan S.S., Shareef H., Ibrahim A.A. (2021). Improved Semi-Empirical Model of Proton Exchange Membrane Fuel Cell Incorporating Fault Diagnostic Feature. J. Mod. Power Syst. Clean Energy.

[37] Nalbant Y., Colpan C.O., Devrim Y. (2020). Energy and Exergy Performance Assessments of a High Temperature-Proton Exchange Membrane Fuel Cell Based Integrated Cogeneration System. Int. J. Hydrogen Energy.

[38] Toghyani S., Baniasadi E., Afshari E. (2019). Numerical Simulation and Exergoeconomic Analysis of a High Temperature Polymer Exchange Membrane Electrolyzer. Int. J. Hydrogen Energy.

[39] Ye L., Jia B., Yin Y. (2015). Modeling and Analysis of HT-PEMFC System Based on Reformed Hydrogen. J. Tianjin Univ. Sci. Technol..

[40] Ay M., Midilli A., Dincer I. (2006). Thermodynamic Modelling of a Proton Exchange Membrane Fuel Cell. Int. J. Exergy.

[41] Li D., Li Y., Ma Z., Zheng M., Lu Z. (2022). Exergetic Performance Coefficient Analysis and Optimization of a High-Temperature Proton Exchange Membrane Fuel Cell. Membranes.

[42] Midilli A., Dincer I. (2009). Development of Some Exergetic Parameters for PEM Fuel Cells for Measuring Environmental Impact and Sustainability. Int. J. Hydrogen Energy.

[43] Sousa T., Mamlouk M., Scott K. (2010). An Isothermal Model of a Laboratory Intermediate Temperature Fuel Cell Using PBI Doped Phosphoric Acid Membranes. Chem. Eng. Sci..

[44] Cheddie D., Munroe N. (2006). Analytical Correlations for Intermediate Temperature PEM Fuel Cells. J. Power Sources.

[45] Olapade P.O., Meyers J.P., Borup R.L., Mukundan R. (2011). Parametric Study of the Morphological Proprieties of HT-PEMFC Components for Effective Membrane Hydration. J. Electrochem. Soc..

[46] Lee W.Y., Kim M., Sohn Y.J., Kim S.G. (2016). Power Optimization of a Combined Power System Consisting of a High-Temperature Polymer Electrolyte Fuel Cell and an Organic Rankine Cycle System. Energy.

[47] Li D., Li S., Ma Z., Xu B., Lu Z., Li Y., Zheng M. (2021). Ecological Performance Optimization of a High Temperature Proton Exchange Membrane Fuel Cell. Mathematics.

[48] Xu B., Li D., Ma Z., Zheng M., Li Y. (2021). Thermodynamic Optimization of a High Temperature Proton Exchange Membrane Fuel Cell for Fuel Cell Vehicle Applications. Mathematics.

[49] Guo X., Zhang H., Zhao J., Wang F., Wang J., Miao H., Yuan J. (2019). Performance Evaluation of an Integrated High-Temperature Proton Exchange Membrane Fuel Cell and Absorption Cycle System for Power and Heating/Cooling Cogeneration. Energy Convers. Manag..

[50] Haghighat Mamaghani A., Najafi B., Casalegno A., Rinaldi F. (2018). Optimization of an HT-PEM Fuel Cell Based Residential Micro Combined Heat and Power System: A Multi-Objective Approach. J. Clean. Prod..

[51] Ay M., Midilli A., Dincer I. (2006). Exergetic Performance Analysis of a PEM Fuel Cell. Int. J. Energy Res..

[52] Chitsaz A., Haghghi M.A., Hosseinpour J. (2019). Thermodynamic and Exergoeconomic Analyses of a Proton Exchange Membrane Fuel Cell (PEMFC) System and the Feasibility Evaluation of Integrating with a Proton Exchange Membrane Electrolyzer (PEME). Energy Convers. Manag..

[53] Nguyen H.Q., Aris A.M., Shabani B. (2016). PEM Fuel Cell Heat Recovery for Preheating Inlet Air in Standalone Solar-Hydrogen Systems for Telecommunication Applications: An Exergy Analysis. Int. J. Hydrogen Energy.

[54] Obara S., Tanno I., Kito S., Hoshi A., Sasaki S. (2008). Exergy Analysis of the Woody Biomass Stirling Engine and PEM-FC Combined System with Exhaust Heat Reforming. Int. J. Hydrogen Energy.

[55] Taner T. (2018). Energy and Exergy Analyze of PEM Fuel Cell: A Case Study of Modeling and Simulations. Energy.

[56] Cohce M.K., Dincer I., Rosen M.A. (2011). Energy and Exergy Analyses of a Biomass-Based Hydrogen Production System. Bioresour. Technol..

